# A case of trigeminal nerve neurosarcoidosis initially presenting with isolated neurological findings mimicking trigeminal schwannoma: a case report and review of the literature

**DOI:** 10.1186/s13256-026-05927-x

**Published:** 2026-03-11

**Authors:** Ahmed Msherghi, Ceylan Altintas Taslicay, Heba Al Qudah, Hamza A. Salim, Sahar Alizada, Muhammed Elhadi, Max Wintermark, Maria Gule-Monroe

**Affiliations:** 1 Department of Neuroradiology, Division of Diagnostic Imaging, The University of Texas MD Anderson Cancer Center, Houston, TX 77030 USA; 2 College of Medicine, Korea University, 145 Anam-Ro, Seongbuk-Gu, Seoul, 02841 Republic of Korea (South Korea)

**Keywords:** Neurosarcoidosis, Sarcoidosis, Trigeminal lesion, Cranial nerves, Meckel’s cave, Non-caseating granuloma

## Abstract

**Background:**

Sarcoidosis is a granulomatous inflammatory disease primarily affecting the lungs. Central nervous system involvement is rare, occurring in approximately 5–10% of cases. Isolated lesions of the cranial nerve, particularly affecting the trigeminal nerve, are even less common. These lesions can closely resemble more well-known tumors, such as trigeminal schwannomas, meningioma, and multiple sclerosis, which can complicate diagnosis when a solitary mass in Meckel’s cave is the initial finding.

**Case presentation:**

We report a case of a 45-year-old white man who presented with several months of right facial discomfort and numbness in the mandibular (V3) distribution consistent with trigeminal neuralgia. Magnetic resonance imaging of the brain revealed an avidly contrast-enhancing lesion in the right Meckel’s cave along the trigeminal nerve. The patient underwent a skull base surgical exploration and resection. Histopathological, immunohistochemistry, and infectious screening of the resected tissue demonstrated noncaseating granulomas with no evidence of neoplastic or infectious etiologies. Lab work of angiotensin-converting enzyme levels and inflammatory markers were within normal limits. Importantly, the patient had presented with isolated trigeminal neurological symptoms, and thoracic abnormalities of mediastinal and hilar calcified lymph nodes with perilymphatic nodules were discovered only during the postoperative systemic evaluation prompted by the histopathological diagnosis. The patient started high-dose corticosteroid therapy following the operation, and his trigeminal pain and neurological symptoms improved substantially; he remained stable for the entirety of the 12-month follow-up duration.

**Conclusion:**

This case highlights the diagnostic challenges of trigeminal nerve lesion. There are no specific imaging features on magnetic resonance imaging that reliably distinguish neurosarcoidosis from tumors such as schwannomas or meningiomas. Therefore, neurosarcoidosis should be included in the differential diagnosis of contrast-enhancing Meckel’s cave masses, even in patients without known systemic sarcoidosis. Tissue biopsy can be invaluable in determining inflammatory lesions and preventing overly aggressive resections. Once diagnosed, neurosarcoidosis is typically managed with high-dose corticosteroids and follow-up, often leading to symptom improvement. Timely histological confirmation and collaborative, multidisciplinary management are essential for achieving correct diagnosis and favorable outcomes.

## Introduction

Sarcoidosis is a multisystem granulomatous disorder of unknown etiology that predominantly affects the lungs and lymphatic system [1]. It is characterized by the formation of noncaseating granulomas in affected tissues, with a global prevalence that varies significantly by geographic region and ethnicity. The highest incidence rates are observed in Northern European populations, particularly in Scandinavian countries and among African Americans in the USA [2, 3].

While neurosarcoidosis is a recognized manifestation, it is relatively rare, occurring in approximately 5–15% of sarcoidosis cases [4]. The pathophysiology involves granulomatous inflammation affecting the central and peripheral nervous systems, with various presentations depending on the anatomical structures [5, 6]. Isolated neurosarcoidosis, particularly involving the trigeminal nerve, is even more uncommon and poses significant diagnostic challenges [7, 8].

Owing to overlapping clinical and radiological features, the condition can mimic other pathologies, such as schwannomas or meningiomas. Diagnostic criteria for neurosarcoidosis have evolved, with the most recent classifications emphasizing the importance of histopathological confirmation whenever possible [9]. Advanced imaging techniques, including magnetic resonance imaging (MRI) with gadolinium enhancement and [^18F]Fluorodeoxyglucose Positron Emission Tomography and CT scan ([^18F]FDG PET/CT) scans, have improved diagnostic accuracy but may still yield nonspecific findings [10]. This case highlights the importance of a multidisciplinary approach in diagnosing and managing such rare presentations.

## Case presentation

The patient, a 45-year-old white male, presented with a 6-month history of severe, episodic facial pain localized to the right side of the face, consistent with trigeminal neuralgia. The pain was described as sharp, stabbing, and triggered by activities such as chewing and speaking. The initial medical history, family history, and physical examination were unremarkable, and there was no prior history of systemic sarcoidosis or autoimmune conditions. At the time of initial evaluation, the patient reported only trigeminal neuralgia symptoms, and no systemic or respiratory complaints were documented. Only after the biopsy confirmed noncaseating granulomatous inflammation did the patient recall a remote episode of shortness of breath, prompting thoracic imaging that subsequently revealed mediastinal and bilateral hilar calcified lymph nodes with perilymphatic nodules. The patient had no history of smoking or occupational exposure to known respiratory irritants.

**Fig. 1:**
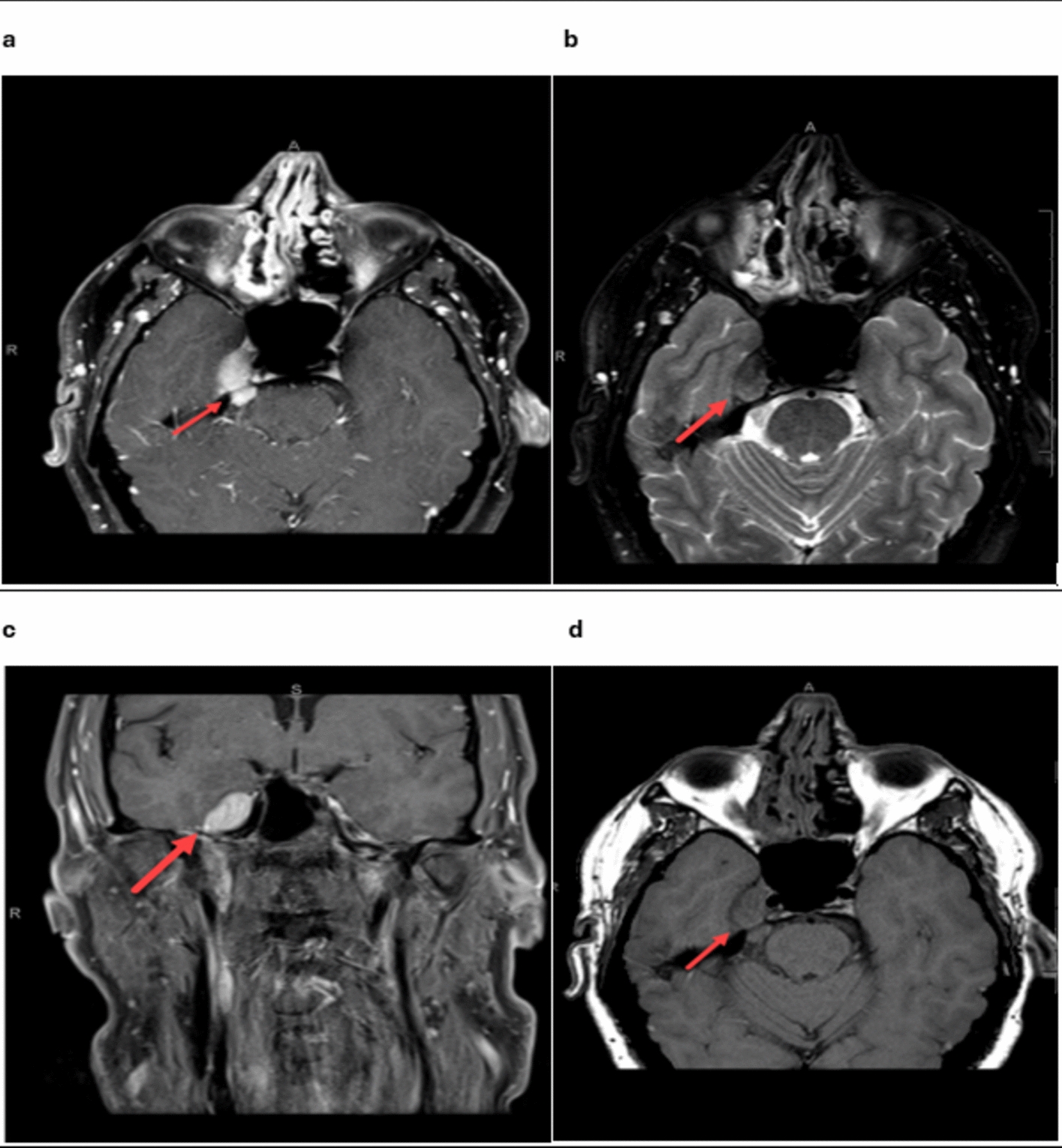
Multisequence magnetic resonance imaging of the brain, with and without intravenous contrast, demonstrates findings consistent with neurosarcoidosis involving the right trigeminal nerve. Pre-operative magnetic resonance images of the skull base, including post-contrast axial T1 (**a**), axial T2 (**b**), post-contrast coronal T1 (**c**), and axial T1-weighted images (**d**). The post-contrast T1 images (**a**, **c**) reveal a homogeneously enhancing, well-defined, mass-like lesion centered on the right Meckel cave (red arrows in images **a** and **c**). The lesion extends posteriorly along the cisternal segment of the trigeminal nerve into the prepontine cistern and abuts the foramen ovale at the skull base without direct extension to the masticator space. On axial T2-weighted images (**b**), the lesion appears mildly T2 hypointense (red arrow)

**Fig. 2:**
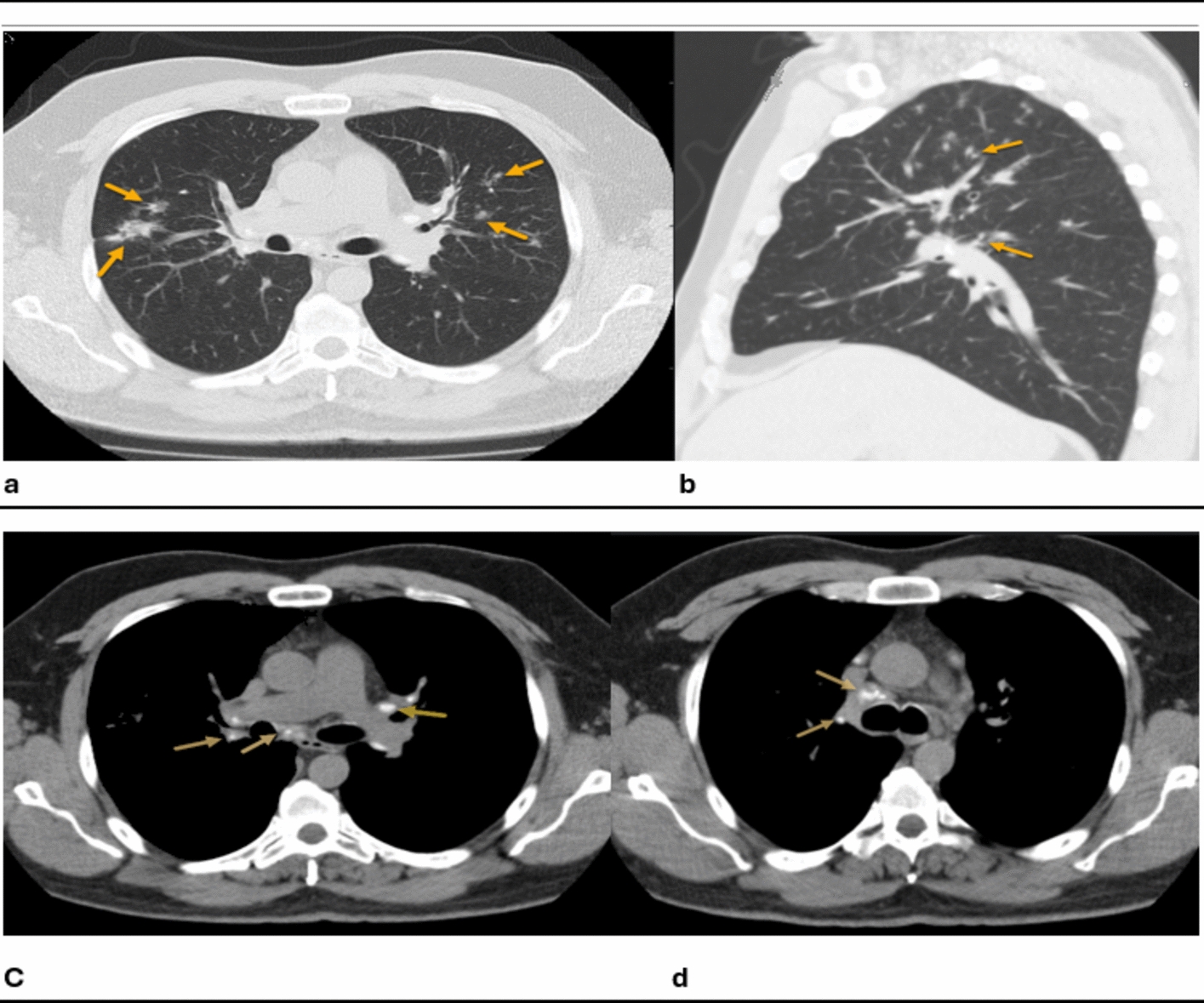
Computed tomography scan of the chest demonstrates radiological features consistent with pulmonary sarcoidosis. Axial (**a**) and sagittal (**b**) chest computed tomography scans with lung window demonstrate diffuse patchy perilymphatic nodules and opacities bilaterally, most notable in the upper lung lobes (the four yellow arrows in image **a** and the two yellow arrows in image **b**). The axial chest computed tomography with mediastinal window (**c**, **d**) shows partially calcified mediastinal and hilar lymph nodes (the three yellow arrows in image **c** and the two yellow arrows in image **d**)

Brain MRI revealed a T1 isointense, T2 mildly hypointense homogeneously enhancing extra-axial lesion centered within the right Meckel’s cave, measuring approximately 2 cm in diameter as shown in Fig. 1. There was an extension posteriorly along the cisternal segment of cranial nerve V3, with the lesion projecting into the prepontine cistern with the abutment of the pons. No extracranial extension along the skull base foramina was detected. The lesion exerted a slight mass effect on the adjacent medial temporal lobe, but with no evidence of parenchymal edema or enhancement. There was no leptomeningeal enhancement. No other intracranial lesions were demonstrated. Chest computed tomography (CT) demonstrated mediastinal and bilateral hilar calcified lymph nodes, with perilymphatic nodular opacities of the lungs, predominantly in the upper lobes (Fig. 2).

Laboratory investigations, including serum angiotensin-converting enzyme (ACE) levels and inflammatory markers, were within normal limits, which did not support a systemic inflammatory or granulomatous process. The patient was initially managed with medical therapy, including gabapentin for neuropathic pain; hydromorphone hydrochloride for breakthrough pain led to minimum to no improvement.

Given the lesion’s homogeneous enhancement, location within Meckel’s cave, and imaging characteristics highly suggestive of a trigeminal schwannoma, surgical exploration and histopathological evaluation were deemed necessary for the brain lesion that was clinically and radiologically suspected to represent trigeminal schwannoma; however, the surgery was not primarily intended for therapeutic decompression. A right cavernous sinus exploration with resection of the trigeminal nerve and ganglion was performed, and the excisional biopsy specimens obtained from the cavernous sinus and trigeminal ganglion were submitted for permanent histopathologic analysis.

Histopathology showed noncaseating granulomatous inflammation of the peripheral nerve with granuloma involving ganglion. The granuloma was composed of epithelioid histiocytes with multinucleated giant cells, including Langhans-type forms and occasional asteroid bodies, accompanied by dense lymphoplasmacytic inflammation.

The history provided and the findings of the special stains for granulomatous infections, Ziehl–Nelsen stain (AFB), Fite-Faraco stain or Wade-Fite stain (Fite), and Grocott–Gömöri’s Methenamine Silver stain (GMS), and the histology for mycobacterial and fungal infections allowed the exclusion of mycobacterial and fungal infections. The granulomas were histiocytic as they showed macrophage granuloma formulation and CD68 (KP1) immunostaining. The IgG4 immunostaining performed failed to show IgG4 related disease. The noncaseating granulomas, with the exclusion of infectious agents with the CD68 foothold, narrow the differential to neurosarcoidosis and exclude granulomatous infections, metastatic lesions, or nerve sheath tumors as possible infectious or neoplastic mimics.

Based on the Zajicek *et al*. and Yang *et al*. diagnostic frameworks, this case qualifies for definite neurosarcoidosis, as the diagnosis was confirmed by direct surgical biopsy and histopathological identification of noncaseating granulomatous inflammation within the nervous system, with no alternative infectious or neoplastic conditions present [11, 12].

Following surgery, the patient was treated initially with intravenous methylprednisolone for acute disease control, followed by a transition to high-dose oral prednisone (approximately 1 mg/kg/day) with a gradual taper over several months, resulting in clinical stabilization and radiologic improvement. The treatment plan was tailored to address both the localized and any potential systemic manifestations of sarcoidosis. No adjunct immunosuppressive therapy was required, as his symptoms and imaging findings improved substantially with corticosteroid treatment alone.

**Fig. 3:**
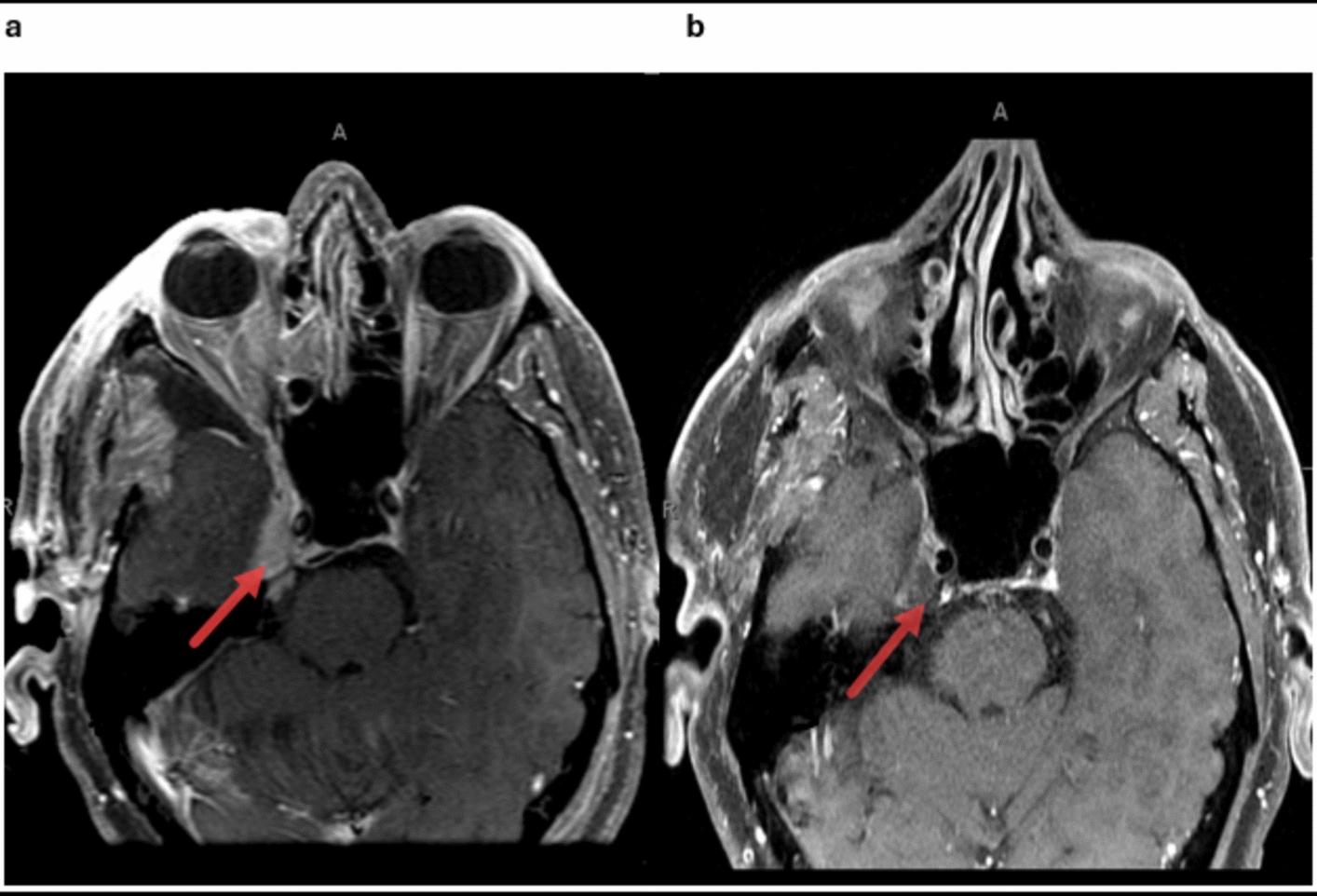
Postoperative and post-treatment brain magnetic resonance imaging with intravenous contrast demonstrates stepwise resolution. Immediate postoperative image (**a**) showing debulking of the tumor in Meckel’s cave with residual tumor extending posteriorly along the cisternal segment of the trigeminal nerve (red arrow). Additional image from the most recent follow-up (**b**) showing near enhancement resolution (red arrow)

The patient demonstrated significant improvement in facial pain and overall quality of life following the initiation of systemic corticosteroid therapy. At the 3-month follow-up, repeat imaging showed a reduction in the lesion size, with no new lesions identified (Fig. 3). The patient reported minimal residual pain, managed effectively with a low dose of gabapentin. Regular follow-up visits were scheduled to monitor for potential recurrence or systemic involvement. At the 1-year follow-up, the patient remained stable, with no evidence of disease progression or new symptoms. This highlights the efficacy of a multidisciplinary approach, including medical and surgical intervention, in managing isolated neurosarcoidosis [1, 8].

## Systematic review

A literature review was conducted in the Medline (PubMed) database from inception to April 2025, searching for studies that conducted among patients with neurosarcoidosis who first presented with sole neurological symptoms. Search terms comprised:

(neurosarcoidosis[Title/Abstract] OR “neurosarcoidosis”[MeSH Terms])

AND

(presentation[Title/Abstract] OR “initial presentation”[Title/Abstract] OR “first presentation”[Title/Abstract] OR “disease onset”[Title/Abstract] OR “initial manifestation”[Title/Abstract] OR “first manifestation”[Title/Abstract] OR “clinical manifestation”[Title/Abstract] OR “early manifestation”[Title/Abstract] OR “early symptom”[Title/Abstract] OR “presenting symptom”[Title/Abstract] OR “presenting feature”[Title/Abstract])

The search term yielded 330 studies, and of these, 10 studies were screened by full text for eligibility and only 3 studies were deemed eligible for enrollment as summarized in Table 1 [13–15].

**Table 1:** Literature review of isolated neurosarcoidosis cases involving trigeminal nerves

Author, year	Age, gender	Clinical presentation	Imaging findings	Confirmed diagnosis	Treatment	Lab findings	Outcome
Quinones-Hinojosa 2003 [13]	33F	Left-sided facial pain and numbness and decreased facial sensation in the V(1)–V(2) distribution	Homogeneously contrast-enhancing, mass centered in Meckel’s cave with extension along the cisternal portion	Brain biopsy	Surgical resection and corticosteroids	NA	Symptoms markedly improved (near-complete resolution)
Lev Bangiyev 2015 [14]	49F	Left-sided headache, left eye itching, face pain, and numbness	Homogeneously enhancing mass involving the cisternal segment of the left trigeminal nerve and extending into Meckel’s cave	Brain biopsy	Surgical resection, corticosteroids, and immunosuppressants	Normal ACE, no mediastinal and bilateral hilar adenopathy	Partial resolution with residual patchy numbness and weakness of temporalis muscle
Lydia Slater 2021 [15]	53F	Right-sided facial numbness, jaw weakness, and one episode of transient diplopia	Homogeneously enhancing mass centered in the right Meckel’s cave	Brain biopsy	Corticosteroids only	Normal ACE, nonspecific mediastinal and bilateral hilar lymphadenopathy	Complete resolution

## Discussion and conclusions

Neurosarcoidosis is a rare but serious manifestation of sarcoidosis involving the central or peripheral nervous system and is often challenging to diagnose owing to its nonspecific clinical and imaging features. Magnetic resonance imaging is the primary imaging modality for detecting neurosarcoidosis, with findings that vary depending on the site of involvement. Typical imaging features include leptomeningeal enhancement, which is seen in up to 40% of cases and is often associated with cranial nerve involvement, particularly the facial and optic nerves. Parenchymal lesions, which may appear as T2 hyperintensities, are another common finding and are typically located in the hypothalamus, brainstem, or periventricular white matter. Spinal cord involvement, presenting as longitudinally extensive T2 hyperintensities, is also frequently observed. Dural thickening and enhancement, hydrocephalus, and pituitary or hypothalamic lesions may also be present. Advanced imaging techniques, such as contrast-enhanced MRI and diffusion-weighted imaging, can help identify granulomatous inflammation and differentiate neurosarcoidosis from other conditions, such as multiple sclerosis or neoplastic processes [9, 16, 17]. The combined radiological features with clinical suspicion are the main initial steps to reach the diagnosis of neurosarcoidosis [16].

However, while suggestive, diagnostic imaging, such as MRI and [^18F]FDG PET/CT scans, often yields nonspecific results. Trigeminal neurosarcoidosis should be considered in the differential of an isolated homogeneously enhancing mass in Meckel’s cave, particularly when other pulmonary or autoimmune findings are present, or the clinical presentation is atypical. These findings are consistent with all previous case reports of isolated neurosarcoidosis (Table 1). In such cases, surgical resection or intracranial biopsy is typically necessary, as histopathological confirmation of noncaseating granulomas remains the gold standard for definitive diagnosis [18]. Furthermore, initiating corticosteroid and immunosuppressive therapy results in marked improvement of neurological symptoms and prevents recurrence of this systemic inflammatory condition.

Our report and Slater *et al*. identified bilateral calcified hilar lymphadenopathy with isolated trigeminal neurosarcoidosis [15]. Furthermore, our patient had pulmonary parenchymal findings on CT. Thoracic imaging with CT should be considered when cranial nerve neurosarcoidosis is part of the differential diagnosis. Sarcoidosis is known to be very avid for [^18F]FDG PET/CT, and whole-body PET imaging may help identify subclinical sites of disease involvement. Furthermore, serum angiotensin-converting enzyme (ACE) levels consistently remained within the normal range during the acute phase in our and previous reports, highlighting the low reliability of serum ACE as a diagnostic marker for isolated neurosarcoidosis of the trigeminal nerve. Previous studies have demonstrated low sensitivity and fair specificity for serum ACE, indicating that while its absence does not exclude sarcoidosis, its presence may support the diagnosis, particularly when the pretest probability is high [4, 16].

In our study and the case reports listed in Table 1, angiotensin-converting enzyme levels in the cerebrospinal fluid (CSF-ACE) were not assessed. Studies have demonstrated that CSF-ACE levels have variable sensitivity (24–66.7%) but high specificity (67.3–95%) for diagnosing neurosarcoidosis. Assessing CSF-ACE levels in cases with a high index of suspicion for isolated neurosarcoidosis of the trigeminal nerve is likely a valuable diagnostic practice. This approach could help distinguish neurosarcoidosis from other differential diagnoses, such as schwannoma and meningioma, given the high specificity of CSF-ACE levels. Furthermore, measuring CSF-ACE levels during lumbar puncture for other laboratory evaluations may facilitate early diagnosis of neurosarcoidosis [19–21].

Differentiating neurosarcoidosis from its imitative entities with isolated neurological findings owing to the complexities it poses in the diagnostic process requires attention. While the clinical presentation and histopathological diagnosis are vital, some particular and diagnostic imaging features will most likely narrow the differential diagnosis owing to the precise and accurate imaging available.

Neuroimaging of neurosarcoidosis involving the trigeminal nerve may be near identical to imaging findings in trigeminal schwannoma, especially with a solitary mass located in Meckel’s cave [14]. Both entities present as avidly enhancing lesions and can extend along the cisternal segment of cranial nerve V as well as through adjacent skull base foramina. Surgical pathology remains the most definitive method in differentiation owing to the lack of definitive and distinguishing imaging features on MRI that differentiate neurosarcoidosis from schwannomas [13]. Diagnosing neurosarcoidosis and excluding neoplastic conditions such as schwannomas requires the presence of noncaseating granulomas in histopathological findings.

Neurosarcoidosis may also mimic meningiomas, especially in cases with dura mater involvement. Dural lesions from sarcoidosis, however, when observed via MRI, are usually marked by T2 signal hyperintensity. This characteristic may be explained by fibrocollagenous tissue development [22]. In some instances, lesions may be presented with T2 hyper- or isointensity, potentially simulating a solid tumor, likely an inflammatory rather than fibrotic process [22]. The functional MRI characteristics of sarcoid granulomas can also be mistaken for those of solid tumors, showing moderate diffusion with rapid initial permeability, and washout on Dynamic Contrast-Enhanced (DCE) MRI (DCE-MRI) perfusion. This substantial overlap suggests the need for tissue sampling and histology for an accurate diagnosis [22].

Future research should prioritize the identification of reliable biomarkers and advanced imaging techniques to enable earlier and more accurate diagnosis, ultimately improving patient outcomes. We strongly encourage conducting high-quality, large-scale case–control studies to establish a standardized approach for managing isolated cranial nerve neurosarcoidosis. Such studies should delineate the roles of laboratory diagnostics, imaging modalities, and interventional procedures in this rare disease entity, providing a comprehensive framework for both diagnosis and treatment.

## Data Availability

All data and reports are available upon request.

## Abbreviations

ACE: Angiotensin-converting enzyme
CD68 (KP1): Macrophage immunomarker
IgG4: Immunoglobin Gamma 4
